# Cycling our way to fit fat

**DOI:** 10.14814/phy2.13247

**Published:** 2017-04-12

**Authors:** Logan K. Townsend, Carly M. Knuth, David C. Wright

**Affiliations:** ^1^ Department of Human Health and Nutritional ScienceUniversity of GuelphGuelphCanada

**Keywords:** Adipose Tissue, AMPK, exercise, mitochondria, PGC‐1*α*

## Abstract

Adipose tissue is increasingly being recognized as a key regulator of whole body carbohydrate and lipid metabolism. In conditions of obesity and insulin resistance mitochondrial content in this tissue is reduced, while treatment with insulin sensitizing drugs such as thiazolidinediones (TZDs) increase mitochondrial content. It has been known for decades that exercise increases mitochondrial content in skeletal muscle and now several laboratories have shown similar effects in adipose tissue. To date the specific mechanisms mediating this effect have not been fully identified. In this review we highlight recent work suggesting that increases in lipolysis and subsequently fatty acid re‐esterification trigger the activation of 5' AMP‐activated protein kinase (AMP) activated protein kinase and ultimately the induction of mitochondrial biogenesis. It is our current view that this pathway could be a unifying mechanism linking numerous systemic factors (catecholamines, interleukin‐6, meteorin‐like) to induction of mitochondrial biogenesis following exercise.

## Introduction

Adipose tissue (AT) has a remarkable ability to expand and accommodate excess energy intake. From an evolutionary standpoint this is beneficial, as it would facilitate the storage of “extra” calories as fat providing the organism with a fuel depot to be tapped into between meals. But nowadays the majority of individuals in Westernized society have ready access to a wide variety of nutrient dense foods. The chronic over‐consumption of foods can overwhelm the storage capacity of AT leading to local (Choe et al. 2016) and systemic (Rosen and Spiegelman 2014) perturbations in carbohydrate and lipid metabolism. Moreover, the important metabolic, endocrine, and inflammatory properties of AT are now intricately linked to the etiology of obesity‐associated insulin resistance and type 2 diabetes (Guilherme et al. 2008). In light of the current surge in the rates of obesity (Flegal et al. 2016) and type 2 diabetes (Smyth and Heron 2006; Roglic and Unwin 2010), interest in AT biology, and how it is perturbed by chronic nutrient excess, is growing.

There is a growing body of evidence linking reductions in AT mitochondria to the development of insulin resistance. For example, in conditions of severe insulin resistance AT mitochondrial proteins are reduced (Choo et al. 2006; Koh et al. 2007), though it should be noted that this is unlikely to be a causal event in the etiology of diet‐induced insulin resistance (Sutherland et al. 2008). Moreover, thiazolidinediones (TZD), a class of insulin‐sensitizing medications, improve systemic glucose homeostasis partially by inducing AT mitochondrial biogenesis (Wilson‐Fritch et al. 2004; Choo et al. 2006). Unfortunately, TZDs are linked to weight gain (Nichols and Gomez‐Caminero 2007), heart attack (Lipscombe et al. 2007), and bladder cancer (Turner et al. 2014) making it important to find alternative means of attaining their benefits, including increased AT mitochondrial content, without the associated risks. This review will specifically focus on evidence that the exercise‐induced re‐esterification of fatty acids plays an important role in mitochondrial biogenesis in AT which contributes to proper AT function and metabolic health.

## Exercise‐Induced Mitochondrial Biogenesis in Adipose Tissue

It has been known for decades that exercise increases skeletal muscle mitochondrial content (Holloszy 1960). Much later, Stallknecht and colleagues (Stallknecht et al. 1991) used a strenuous exercise protocol of 6 h/d of swim training for 12 weeks to demonstrate, for the first time, that exercise can increase mitochondrial enzyme activity in white AT depots of rats. These findings were later confirmed by Sutherland et al. (2009) who reported increases in several mitochondrial proteins in white AT from rats following 4 weeks of daily (2 h/day) swim exercise. While these studies clearly show increased mitochondrial proteins in AT, the extremely strenuous nature of the exercise calls into question the clinical relevance of these findings. However, recent work using forced treadmill running (Xu et al. 2011) and voluntary wheel running (Stanford et al. 2015; Peppler et al. 2016) also report increased mitochondrial proteins in AT.

Taken together, these data support that exercise training increases mitochondrial enzyme content and activity in AT, much like skeletal muscle (Little et al. 2011; Wright 2014). Unfortunately, despite the growing appreciation for the importance of AT mitochondria there is a dearth of research exploring the mechanistic regulation of mitochondrial biogenesis in AT. It has been our working hypothesis that the exercise‐induced increase in lipolysis and re‐esterification are at least partly responsible for mitochondrial biogenesis in AT.

## Regulation of Lipolysis and Re‐Esterification

Lean adults store ~80 000 kcal as triglycerides (TG) in AT (Horowitz 2003). Prolonged exercise relies heavily on the breakdown of these TGs into fatty acids (FA) and the transport of these FAs to other tissues to be oxidized for energy. Indeed, even during low intensity exercise (25% *V*O_2max_) the rate of lipolysis increases up to fivefold compared to rest (Wolfe et al. 1990).

**Figure 1:**
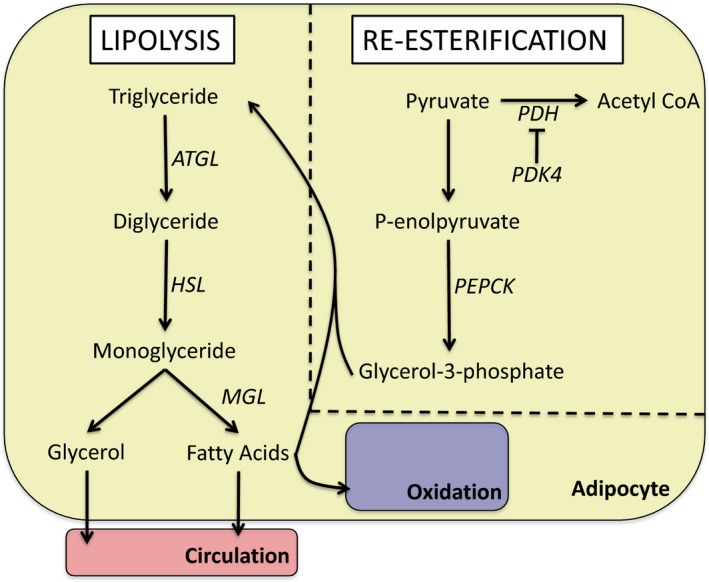
The biochemical pathways regulating lipolysis and fatty acid re‐esterification. Triglycerides are broken down to glycerol and fatty acids by the sequential actions of adipose triglyceride lipase, hormone sensitive lipase and monolglyceride lipase. Re‐esterification involves the conversion of pyruvate to glycerol‐3‐phosphate by phosphoenolpyruvate carboxykinase. This process is aided by pyruvate dehydrogenase kinase 4 which inhibits the activity of the pyruvate dehydrogenase complex, thereby shuttling pyruvate away from acetyl‐CoA and towards re‐esterification.

Lipolysis is the process of hydrolyzing TG to FAs and glycerol via the sequential actions of adipose triglyceride lipase (ATGL), hormone‐sensitive lipase (HSL), and monoglyceride lipase (as reviewed in (Duncan et al. 2007)). The liberated FAs have three subsequent fates: oxidized within the adipocyte, released into systemic circulation, or re‐esterified back into TG. A very small percentage is oxidized (<0.5%) while the rest is divided nearly evenly between release and re‐esterification, at least in the fed state (Wang et al. 2003) (Fig. 1).

Importantly, the absolute rate of re‐esterification increases linearly and proportionately with lipolysis (Vaughan 1962; Reshef et al. 1970; Brooks et al. 1982). Lipolytic activity increases dramatically during exercise and while it may seem counterintuitive to simultaneously increase re‐esterification during exercise, as it would limit the availability of FA to active tissues, this may serve to limit any lipotoxic effects of greatly elevated circulating FAs (Mottillo et al. 2012). Whatever the teleological explanation, TG‐FA recycling is ~4‐times greater in endurance trained athletes compared to sedentary age‐matched controls (Romijn et al. 1993) and re‐esterification is blunted in FAT/CD36 knockout (Wan et al. 2014) and aged mice (Mennes et al. 2014), two models of reduced lipolysis, supporting the link between lipolysis and re‐esterification in AT.

Re‐esterification depends on the adequate provision of glycerol‐3‐phosphate (G3P) that serves as the TG backbone (Fig. 1). Glyceroneogenesis is the main source of G3P in AT due to the limited expression of glycerol kinase (Leroyer et al. 2006) and low glucose uptake under fasting conditions (Nye et al. 2008). Two enzymes play important roles in regulating glyceroneogenesis, namely pyruvate dehydrogenase kinase 4 (PDK4) and phosphoenolpyruvate carboxykinase (PEPCK). PDK4 inhibits the pyruvate dehydrogenase complex, thereby blocking the conversion of pyruvate to acetyl‐CoA and shuttling pyruvate toward G3P (Cadoudal et al. 2008). At the same time, PEPCK catalyzes the decarboxylation of oxaloacetate to form phosphoenolpyruvate, which will ultimately be converted into G3P. Overexpression of PEPCK in AT of mice increases FA re‐esterification (Franckhauser et al. 2002) whereas the pharmacological inhibition of PDK4 reduces pyruvate incorporation into TGs (Cadoudal et al. 2008), demonstrating the importance of these enzymes in regulating glyceroneogenesis.

**Figure 2:**
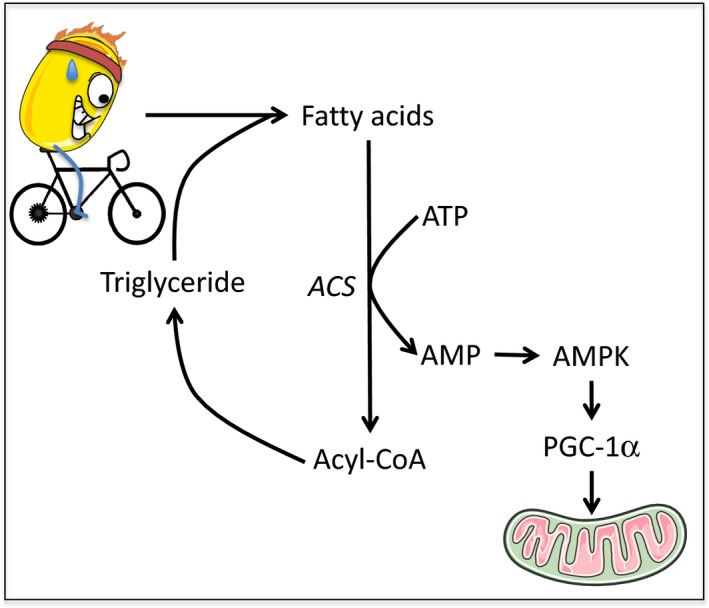
Exercise drives the breakdown of triglyceride molecules into fatty acids. Approximately half of these fatty acids are converted to acyl‐CoA by acyl‐CoA synthetase in a process that produces AMP. Increased AMP can activate AMPK, which would then drive PGC‐1*α* expression and stimulates mitochondrial biogenesis in AT.

Importantly, whereas lipolysis does not consume energy, re‐esterification requires FAs to be acetylated by acyl‐CoA synthetase in a process that consumes ATP and generates AMP‐activated protein kinase (AMP) (Gauthier et al. 2008) (Fig. 2). More specifically, two ATP molecules are required for every FA that is acylated, for a total of 7–9 ATP for the synthesis of a TG molecule, depending on the origin of G3P (Gauthier et al. 2008). In response to lipolytic hormones this process is possibly the greatest consumer of ATP in adipocytes (Rognstad and Katz 1966).

**Figure 3:**
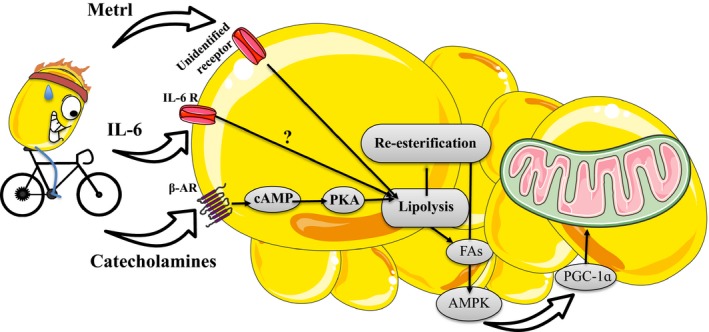
Exercise stimulates secretion of catecholamines, interleukin‐6, and meteorin‐like (metrl). Catecholamines bind to *β*‐adrenergic receptors on the adipocyte to stimulate lipolysis through a PKA‐mediated pathway. IL‐6 stimulates lipolysis, though speculation remains regarding the precise mechanisms. Metrl indirectly drives lipolysis by modulating the secretion of catecholamines from adipose tissue macrophages. These factors activate lipolysis and consequently re‐esterification. This increases AMP‐activated protein kinase activity, the expression of PGC‐1ɑ and mitochondrial biogenesis. Figure clipart provided by Servier Medical Art (www.servier.com).

## AMPK

5′AMP‐activated protein kinase (AMPK) is an energy‐sensing enzyme that responds to an increased AMP:ATP ratio. AMPK is a heterotrimeric protein consisting of a catalytic *α* subunit in addition to *β* and *γ* regulatory subunits (Long and Zierath 2006). The *α* and *β* subunits are each encoded by two genes (*α*1 and *α*2 or *β*1 and *β*2) while the *γ* subunit is encoded by three genes (*γ*1, *γ*2, and *γ*3) (Long and Zierath 2006). ATP turnover can increase >100‐fold in skeletal muscle during exercise compared to rest (Gaitanos et al. 1993) making it unsurprising that AMPK is an important metabolic regulator in this tissue. For example, daily dosing of rats with aminoimidazole‐4‐carboxamide ribonucleotide (AICAR) induces mitochondrial gene expression in skeletal muscle (Winder et al. 2000), potentially through its ability to activate AMPK. On the other hand, muscle specific knockout of AMPK greatly reduces mitochondrial content (O'Neill et al. 2011), demonstrating the importance of AMPK in mitochondrial regulation in skeletal muscle.

It is becoming increasingly evident that AMPK has important functions in AT (Long and Zierath 2006; Steinberg and Kemp 2009). Much like in skeletal muscle, AMPK activity in AT appears to be secondary to an increased AMP:ATP ratio. Gauthier and colleagues (Gauthier et al. 2008) demonstrated that the pharmacological activation of lipolysis in 3T3‐L1 adipocytes increased the phosphorylation of AMPK and its downstream target acetyl‐CoA carboxylase (ACC). Moreover, when adipocytes were treated with orlistat, a general lipase inhibitor, lipolysis and AMPK activation were reduced ~50% (Gauthier et al. 2008). Finally, adipocytes were incubated with isoproterenol, to activate lipolysis, with or without triacsin C, an acyl‐CoA synthase inhibitor that impairs re‐esterification. Co‐incubation with triacsin C blunted the isoproterenol‐induced increase in the AMP:ATP ratio and the phosphorylation of AMPK and ACC (Gauthier et al. 2008). Collectively, these data support the notion that AMPK activation in AT is secondary to an increase in the AMP:ATP ratio and that this decreased energy state appears to be due, at least in part, to the acylation of the fatty acids for re‐esterification (Gauthier et al. 2008).

These findings could partially explain recent work from our laboratory showing that reductions in lipolysis are associated with attenuated AMPK activation. By comparing young (11 week) and old (24 mo) mice we observed that older mice expressed lower protein content/phosphorylation of ATGL, HSL, and PEPCK, indicative of diminished lipolysis and re‐esterification, respectively (Mennes et al. 2014). Concomitant with this we observed diminished AMPK phosphorylation and mitochondrial proteins in epididymal AT of old mice (Mennes et al. 2014). Interestingly, in this project we also observed a significantly increased FA/glycerol ratio, a marker of diminished re‐esterification, which could explain the reduced AMPK and mitochondrial enzymes, at least with aging. Taken together, this might ultimately contribute to the various metabolic abnormalities associated with aging, such as insulin resistance (López‐Otín et al. 2013).

AMPK can modulate the activity of PGC‐1*α* in skeletal muscle (Jäger et al. 2007). Similarly, incubation of adipocytes with the non‐specific AMPK agonist AICAR significantly increased the expression of PGC‐1*α* (Gaidhu et al. 2009), suggesting that AMPK can also modulate PGC‐1*α* expression in white AT. This relationship may explain why we recently observed diminished PGC‐1*α* expression and mitochondrial markers in epididymal AT of AMPK*β*1 knockout mice compared to wildtype animals (Wan et al. 2014). Moreover, in response to ex vivo treatment with norepinephrine or CL316,243, both potent *β*‐adrenergic activators, there was a markedly blunted induction of PGC‐1*α* expression in epididymal AT cultures from AMPK*β*1 knockouts (Wan et al. 2014).

Although there is convincing evidence that lipolysis and AMPK are important mediators of AT mitochondrial biogenesis, work from Granneman's lab demonstrates otherwise (Mottillo and Granneman 2011). This group observed that the acute treatment of 3T3‐L1 adipocytes with CL316,243, a *β*‐3 adrenergic agonist, increased PGC‐1*α* expression, but impeding FA release by pharmacologically blocking HSL or knocking down ATGL actually potentiated the induction of PGC‐1*α*. Additionally, when HSL was pharmacologically inhibited in vivo by BAY 59‐9435 prior to treatment with CL316,243 there was a > 5‐fold potentiation in the expression of PGC‐1*α*, NOR‐1 and UCP‐1 in mouse epididymal AT compared to CL 316,243 treated controls (Mottillo and Granneman 2011). Despite AMPK activity not being assessed in this project, the results are difficult to reconcile with other work showing that AMPK activation is diminished when the release of FA is reduced (Watt et al. 2006; Gauthier et al. 2008), although only mRNA was measured, which could potentially explain the conflicting results.

In light of these findings we analyzed the relationship between lipolysis, AMPK, and PGC‐1*α* expression in ATGL knockout mice and adipocytes treated with ATGListatin, an ATGL inhibitor (MacPherson et al. 2016). We observed that CL316,243‐induced AMPK activity was blunted in both models but PGC‐1*α* expression was intact in both ATGL deficient mice and 3T3‐L1 adipocytes treated with ATGListatin (MacPherson et al. 2016). These findings seem to show that, at least under conditions of reduced FA release in the presence of a robust activation of PKA, AMPK is not required for the *β*‐adrenergic‐mediated induction of PGC‐1*α* and mitochondrial biogenesis. As FAs have been shown to directly attenuate the expression of PGC‐1*α* in adipocytes (Gao et al. 2010) we speculate that the blunted release of FAs relieves an inhibitory effect of FAs on PGC‐1*α* that may mask the reduction in AMPK activity.

## PGC‐1α

PGC‐1*α* is a master regulator of mitochondrial biogenesis (Puigserver et al. 1998; Rohas et al. 2007). The overexpression of PGC‐1*α* in human subcutaneous adipocytes increased mitochondrial enzyme expression (Tiraby et al. 2003) while the AT‐specific deletion of PGC‐1*α* in mice decreased expression of mitochondrial genes (Kleiner et al. 2012). At the same time, insulin‐resistant rodents (Valerio et al. 2006), obese individuals (Semple et al. 2004), and type 2 diabetics (Bogacka et al. 2005) all express diminished AT PGC‐1*α*. Additionally, TZD‐induced AT mitochondrial biogenesis is accompanied by increased PGC‐1*α* expression (Bogacka et al. 2005). These data show that AT PGC‐1*α* plays an important role in systemic glucose homeostasis, conceivably via its role in AT mitochondrial biogenesis.

PGC‐1*α* is important for exercise‐induced mitochondrial biogenesis in skeletal muscle (Safdar et al. 2011) and, although far less researched, we have shown that exercise training also induces PGC‐1*α* mRNA expression in AT. Immediately following 2 h of swimming, PGC‐1*α* mRNA expression was significantly elevated in epididymal and retroperitoneal AT compared to sedentary rats (Sutherland et al. 2009). Performing this protocol daily for 4 weeks significantly increased AT mitochondrial content, suggesting that repeated acute activation of PGC‐1*α* could contribute to exercise‐induced mitochondrial biogenesis. Indeed, at the protein level, we have recently observed significantly increased PGC‐1*α* content in epididymal and inguinal AT depots following 10 weeks of voluntary wheel running (Peppler et al. 2016).

Similar to the activation of AMPK, the induction of PGC‐1*α* could be secondary to increases in FA re‐esterification. Utilizing AT cultures from FAT/CD36 knockout mice that have attenuated FA uptake and lipolysis, we observed an increased FA:glycerol ratio and ~50% reduction in PEPCK mRNA expression (Wan et al. 2013), indicative of reduced re‐esterification. Interestingly, in these knockout mice we also observed diminished expression of PGC‐1*α* (Wan et al. 2013) supporting our hypothesis that re‐esterification can activate PGC‐1*α* and mediate AT mitochondrial biogenesis. One attractive explanation for these observations is that the reduced FA uptake and lipolysis led to reduced re‐esterification. Attenuated re‐esterification lessened the cellular stress resulting from FA acetylation, decreasing the activation of AMPK (Gauthier et al. 2008), an enzyme known to regulate PGC‐1*α* (Jäger et al. 2007) and mitochondrial biogenesis in AT (Wan et al. 2014).

## Catecholamines, IL‐6 and Other Potential Mediators

We hypothesize that the rise in circulating catecholamines during exercise activates lipolysis and increases FA re‐esterification leading to the activation of AMPK and the subsequent induction of PGC‐1*α*. Given this proposed pathway, it seems likely that any stimuli that activate lipolysis should therefore induce mitochondrial biogenesis in AT.

It is now recognized that skeletal muscle is an active endocrine organ and it has been postulated that contraction‐stimulated increases in the secretion of muscle‐derived signaling peptides or myokines could mediate some of the beneficial effects of exercise on AT metabolism. For example, much recent work has focused on interleukin 6 (IL‐6). Exercise can stimulate IL‐6 secretion from skeletal muscle (Ostrowski et al. 1998) while IL‐6 infusions have been shown to increase indices of whole body lipolysis (van Hall et al. 2003). It should be noted however that the direct effects of IL‐6 in stimulating lipolysis in cultured adipocytes (van Hall et al. 2003) or AT (Wan et al. 2012) are muted compared to that of catecholamines. That being said, there is accumulating evidence to suggest the involvement of IL‐6 in the exercise‐mediated adaptations in AT. For example, IL‐6 injections (Knudsen et al. 2014) or treating cultured AT with IL‐6 results in increases in the expression of UCP‐1 and PGC‐1*α*, respectively. Moreover, exercise training‐induced increases in UCP‐1 in inguinal white AT are absent in whole body IL‐6 deficient mice (Knudsen et al. 2014). While these findings hint at the possibility of a muscle‐IL‐6‐AT signaling axis recent work has shown intact exercise‐induced increases in circulating IL‐6 from muscle specific IL‐6 knockout mice (Gudiksen et al. 2016) providing evidence that muscle is not the sole producer of IL‐6 during exercise and/or the recovery from it.

Our working model hypothesizes that increase in circulating catecholamines could serve as a trigger for exercise‐induced AT remodeling. Intriguing recent data from Spiegelman's group provides evidence that localized increases in catecholamine production within AT could also play a role in mediating the effects of exercise (Rao et al. 2014). These authors discovered that the overexpression of PGC‐1*α* or damaging downhill running increased the secretion of a novel myokine, meteorin‐like (metrl). Increased circulating metrl was reported to result in the browning of white AT through a mechanism involving an M2 macrophage‐dependent increase in localized catecholamine production (Nguyen et al. 2011; Rao et al. 2014). These findings highlight the importance of autocrine/paracrine factors triggered by muscle‐based signals in the exercise‐mediated regulation of adipose tissue metabolism. It is yet to be determined if metrl and subsequent increases in catecholamines signal through AMPK to induce a browning of white AT.

## The Relevance of Lipolysis and AMPK Activation to the Browning of Adipose Tissue

Recent work has identified the presence of brown adipose tissue in humans (Cypess et al. 2009). Moreover, it has been shown that white adipose tissue can take on brown adipose tissue like characteristics, that is, “browning” (Wu et al. 2012). These findings have garnered much recent attention for their potential role in treating obesity and diabetes (Nedergaard and Cannon 2014; Sidossis and Kajimura 2015). A hallmark feature of brown adipose/browned AT is the presence of uncoupling protein‐1 (UCP‐1). This protein uncouples respiration from ATP synthesis and generates heat. In addition, more recent work has provided evidence for UCP‐1 impacting glucose homeostasis independent of changes in adiposity (Winn et al. 2017). Importantly, both AMPK (Mottillo et al. 2016) and PGC‐1*α* (Puigserver et al. 1998; Boström et al. 2012) are important drivers of AT browning and thermogenesis. Moreover, catecholamines (Himms‐Hagen et al. 1994), IL‐6 (Knudsen et al. 2014), and metrl (Rao et al. 2014) have all been linked to the browning of white AT. Thus, it is appealing to speculate that the current hypothesis regarding the regulation of mitochondrial biogenesis may extend to the browning of white AT.

## Closing Remarks

The obesity epidemic has ushered in a growing appreciation for AT as a central control point in the regulation of systemic carbohydrate and lipid metabolism. Unfortunately, as opposed to the mechanisms regulating skeletal muscle mitochondrial biogenesis, relatively little is known about AT. Here, we have proposed a potential mechanism whereby re‐esterification is activated in response to exercise, driving the activation of AMPK that enhances the expression of PGC‐1*α*, a chief regulator of mitochondrial biogenesis. This process is primarily activated by catecholamines but additional factors, such as the myokines IL‐6 and meteorin‐like, are emerging as important contributors (Fig. 3). More work into these ideas will ultimately provide a greater understanding of the pathologies to which AT directly contributes, including type 2 diabetes, and potentially lead to new therapeutic targets.

## Conflict of Interest

None declared.
